# A Randomized-Controlled Trial of Computer-based Prevention Counseling for HIV-Positive Persons (HPTN 065)

**DOI:** 10.4172/2155-6113.1000714

**Published:** 2017-07-26

**Authors:** Laura A. McKinstry, Allison Zerbe, Brett Hanscom, Jennifer Farrior, Ann E. Kurth, Jill Stanton, Maoji Li, Rick Elion, Jason Leider, Bernard Branson, Wafaa M. El-Sadr

**Affiliations:** 1 Statistical Center for AIDS Research and Prevention, Fred Hutchinson Cancer Research Center, Seattle, USA; 2 ICAP at Columbia University, Mailman School of Public Health, New York, NY, USA; 3 FHI 360, Durham, North Carolina, USA; 4 Yale School of Nursing, New Haven, CT, USA; 5 District of Columbia District of Health STD/HIV Research Program, Washington, USA; 6 North Bronx Health Care Network, New York, NY, USA; 7 Scientific Affairs LLC, Atlanta, Georgia, USA

**Keywords:** HIV prevention, Prevention for positives, Technology-based intervention, Computer-delivered counseling

## Abstract

**Objective:**

Decreasing the risk of HIV transmission from HIV-positive individuals is an important public health priority. We evaluated the effectiveness of a computer-based sexual risk reduction counseling intervention (CARE+) among HIV-positive persons enrolled in care.

**Methods:**

HIV-positive eligible participants (N=1075) were enrolled from 11 care sites in the Bronx, NY and Washington, DC and randomized 1:1 to either a tablet-based self-administered CARE+ intervention or standard of care (SOC). The primary outcome was the proportion of participants reporting any unprotected vaginal/anal sex at last sex, among all partners, HIV-negative or HIV-unknown-status partners and for primary and non-primary partners.

**Results:**

At baseline, 7% of participants in both arms reported unprotected sex with an HIV-negative or HIV-unknown-status partner, while 13% in the CARE+ arm and 17% in the SOC arm reported unprotected sex with any partner. Most participants (88%) were on antiretroviral therapy (ART) at baseline. There was no significant difference in changes over time in unprotected vaginal/anal sex between the CARE+ and SOC arms for any partners (p=0.67) or either HIV-negative or HIV-unknown-status partners (p=0.40). At the Month 12 visit, most participants (85%) either strongly agreed or agreed that computer counseling would be a good addition to in-person counseling by a provider.

**Conclusion:**

The CARE+ intervention was not effective at reducing sexual risk behaviors among HIV-positive patients in care, most of whom were on ART. Further research may be warranted around the utility of computer-based counseling for HIV prevention.

## Introduction

In the United States, there are more than 1.2 million persons living with HIV (PLWH), with an estimated 39,513 new diagnoses in 2015 [1]. Although most persons who are aware of their positive HIV status reduce risky sexual behaviors [2], for some PLWH, it may be difficult to achieve and sustain safer sexual behaviors [3–5] for numerous reasons. For example, some may believe that condom use reduces sexual pleasure while others may not have the confidence to consistently practice safer sex. Some PLWH may practice unsafe sex only with partners known to be HIV-positive, but the possibility of transmitting and acquiring different HIV strains still exists. While antiretroviral therapy (ART) can substantially reduce sexual HIV transmission [6,7], not all HIV-positive individuals in clinical care are effectively virally suppressed [8]. Thus, specific prevention efforts for PLWH remain critical to preventing the spread of HIV. There is some evidence that “prevention for positives” (PfP) interventions effectively decrease risky sexual behaviors [9–11].

Health care providers can play an important role in helping PLWH reduce risky sexual behaviors and maintain safe sex practices [10]. However, evidence indicates that providers often fail to discuss safe sex practices [12,13]. Despite CDC recommendations and inclusion in clinical guidelines of risk reduction counseling for PLWH [14,15], data on provider-patient communication demonstrate that fewer than half of HIV-positive individuals in care received HIV/sexually transmitted infections (STI) prevention counselling from their health care provider and 39% of PLWH reporting risky sexual behavior did not receive any risk-reduction counseling [16]. Conduct of risk assessments and risk interventions can vary: providers are more likely to provide prevention counseling to newly-diagnosed HIV-positive patients than to established patients [17,18]. Common barriers cited to the conduct of risk assessments and risk-reduction interventions include limited time and/or staff, size of clinic, competing priorities, lack of training to conduct risk-reduction counseling, and discomfort talking about risk behaviors [17–20]. The use of technology-based interventions delivered via computer, tablet or smart phone might help alleviate some of these barriers.

Technology-based health interventions have been used across a number of health issues including autism, smoking cessation and obesity [21–23]. Computer-delivered self-administered questionnaires can help reduce social desirability bias when reporting sexual behaviors and have been found to be acceptable among different populations [24,25]. Specific computer-based interventions for HIV have been shown to improve adherence to ART [26,27] and reduce HIV transmission risk [28–32]. Kurth et al. found that the CARE+ computer-based prevention counseling intervention for HIV-positive individuals was both acceptable and feasible amongst inexperienced computer users [33,34]. Additionally, in a randomized study conducted among a largely men who have sex with men (MSM) population, the CARE+ intervention was found to improve ART adherence and rates of viral suppression as well as reduce risky sexual behavior [35].

The HPTN 065 study, conducted by the HIV Prevention Trials Network (HPTN), examined the feasibility of a test, link-to-care, plus treat strategy for HIV prevention in the Bronx, NY and Washington, DC. The study included 5 components: expanded HIV testing, evaluation of financial incentives for encouraging linkage to care and viral suppression [36], prevention for positives (PfP) and patient and provider surveys. The overall design of the HPTN 065 study is described in detail elsewhere [37]. We report on the PfP component, a two-arm, individually randomized, study that evaluated a modified computer-based prevention counseling intervention (CARE+ PfP) for HIV-positive persons in care to determine its effect on reducing the number of self-reported episodes of unprotected sex.

## Methods

### Sites

Between January and December 2013, HIV-positive men and women receiving care at 11 participating HIV care sites (6 in Washington, DC and 5 in the Bronx, NY) were recruited for the PfP component. These 11 sites, a subset of the 39 HIV care sites that participated in other components of the overall HPTN 065 study, were chosen based on their willingness to participate in this component of the study. Participating sites included 6 hospitals (2 university affiliated hospitals, 2 non-university affiliated hospitals and 2 VA facilities), 3 community health centers and 2 private medical practices. The number of HIV-positive patients enrolled in care at these sites ranged from 500 to 3000. During the course of the study, one site in NY was terminated due to inability to perform.

### Participants

Patients were eligible for enrollment if they were able to consent for HIV care according to New York State or Washington, DC law, were receiving care at the selected study sites, had attended the clinic one or more times in the last seven months, were able to understand either spoken English or Spanish, were able to provide informed consent, were not participating in another study focusing on HIV prevention for positives and did not have a history or evidence of altered mentation, inebriation or substance use that would interfere with study participation. In an effort to allow sites to implement the study without adversely affecting their practices or requiring additional resources, each site developed and implemented site-specific recruitment procedures. Informed consent was obtained from all individual participants included in the study. Participants were financially compensated for their time; payment amounts differed by site but ranged from $10–$25 per visit, enough to cover transportation costs. The study was approved by the local institutional review boards (IRB) affiliated with each site or by a central IRB. All procedures performed in the study were in accordance with the ethical standards of the institutional and/or national research committee and with the 1964 Helsinki declaration and its later amendments or comparable ethical standards.

### Intervention

Participants were randomized 1:1 using a blocked randomization scheme to either the intervention arm (receiving the tablet-based self-administered CARE+ PfP intervention plus standard of care (SOC) HIV prevention services) or to the control arm (receiving only SOC HIV prevention services). Standard of care services were those routinely provided at the sites, such as provider-initiated counseling regarding condom use and safe sex practices, targeted HIV-prevention counseling for high-risk individuals, STD testing and referrals, and HIV prevention literature/hand-outs. Participants in both arms took the same tablet-based HIV risk behavior questionnaire.

The intervention, known as “CARE+” (Computer Assessment and Rx Education for HIV-positive people), is a custom Windows application running on a touchscreen tablet PC, equipped with headphones and connected via Wi-Fi network to a database on a secure local server. It was designed to be administered in the waiting room at the time of a clinic visit. The intervention content was based on several theoretical frameworks including an information-motivation-behavioral skills (IMB) model (used to inform importance and confidence scales related to HIV transmission risk reduction), the transtheoretical model of change (e.g. used to inform questions and messages related to condom use), social cognitive behavioral theory (e.g. used to inform content for the videos where peers demonstrate healthy sexual behavior practices), and motivational interviewing (e.g. used to inform tailored feedback related to ambivalence of changing behavior and commitment to changing). The content has been described in detail elsewhere [35] For this study, the original CARE+ tool was used but with a slight modification; the ART adherence/viral suppression component was removed from the original tool before being used in the PfP component of HPTN065 (the HIV risk reduction component of the original CARE+ tool was not changed). The ART adherence/viral suppression items were removed since the larger HPTN065 study had a separate component of the study focused solely on financial incentives for viral suppression. This revised CARE+ software was pilot tested with the intended study population; software bugs were fixed and minor formatting changes were made.

Participants completed their assigned tablet-based session at baseline and months 3, 6, 9 and 12, with study visits scheduled on the same day as regularly scheduled clinic visits whenever possible. For SOC arm participants, the tablet portion of the sessions consisted of only the HIV risk behavior questionnaire. For those in the intervention arm, the tablet-based session was more detailed. Prior to the start of this same risk behavior questionnaire that both arms completed, intervention arm participants selected a CARE+ avatar that audio-narrated all text and questions. Based on responses to the risk behavior questionnaire, the tool summarized what things the participant had been doing to stay healthy, such as using condoms regularly with all sex partners, and then listed things the participant might want to work on, such as having fewer sex partners, in order to reduce their HIV risk. Intervention participants could then choose to watch skills-building videos (video topics included HIV disclosure, safer sex and condom use negotiation). The intervention arm participants then created a personalized risk-reduction plan. At the end of each session, intervention arm participants could opt to print out their risk reduction plan as well as referrals for STI, suicide prevention, domestic violence and/or sexual assault services, as indicated. At session completion, the tablet was returned to the site staff member and an alert message would appear on the tablet screen if participants’ responses indicated intimate partner violence, depression or suicidal ideation. Although not a focus of the intervention, the site would assist and support such participants, as needed, and ensure referrals for appropriate care were made.

Participants in both the intervention and SOC arms had viral load assessed at baseline and at each follow-up visit. At the baseline and Month 12 sessions, all participants also completed a separate Patient Survey regarding HIV care and prevention that included questions about the use and acceptability of computer-delivered counseling interventions.

### Outcome measures

The primary outcome, collected via the tablet-based HIV risk behavior questionnaire, was the proportion of participants who reported unprotected vaginal or anal sex the last time they had sex. Participants were asked to distinguish whether unprotected sex occurred with a primary (e.g. regular) partner or with non-primary (e.g. casual) partners. Secondary outcomes, also collected via the tablet-based HIV risk behavior questionnaire, included the proportion of participants who reported unprotected vaginal or anal sex at last sex with HIV-negative or HIV-unknown-status partners, evaluated for all partners and stratified for primary and non-primary partners, and the frequency of any unprotected vaginal or anal sex in the previous three months with non-primary partners.

Viral load, collected via chart review, was also evaluated; “detectable viral load” was defined as greater than 50 copies per mL. Regarding the acceptability of computer-delivered counseling interventions, participants were asked in the Patient Survey to state how strongly they agreed or disagreed with the following statements: a) Computer counseling on HIV prevention would be a good addition to counseling given in person by a provider; b) For many HIV positive people a computer can be as good as a real person in providing counseling to prevent the spread of HIV; and c) It is easier to be honest when answering questions on the computer than it is when answering the same questions in person with a provider.

The outcome measures used in this study differed slightly from those used in previous studies of CARE+ [35]. To address these differences, we performed a secondary analysis using the risk measure defined in the Kurth et al. study [35,38]. The risk measure used in that study was a ‘transmission risk’ composite measure comprised of two elements: 1) condom problems in the last 3 months with any partner and 2) sex without a condom at last sex in the last 3 months.

### Statistical methods

Baseline and follow-up data for all participants who completed at least one follow-up visit were included in the primary analyses. Primary and secondary endpoint data, as well as the viral load data, were modeled using logistic regression and generalized estimating equation (GEE) methods for correlated data, with no adjustment for covariates. Errors in study-arm allocation and intervention delivery occurred for 17 participants due to both user and software errors. These errors resulted in 8 participants receiving the intervention when they should not have and 9 participants not receiving the intervention when they should have. Following intent-to-treat principles, these 17 subjects with randomization discrepancies were analyzed in the arm to which they were originally assigned. Data analyses were performed using SAS 9.4 and R 3.2.0. The trial was initially designed to enroll 522 people per arm, which would provide 90% power to detect a decrease from 11% to 8% in proportion of patients reporting any unprotected vaginal or anal sex at a given visit during the study.

## Results

**Figure 1:**
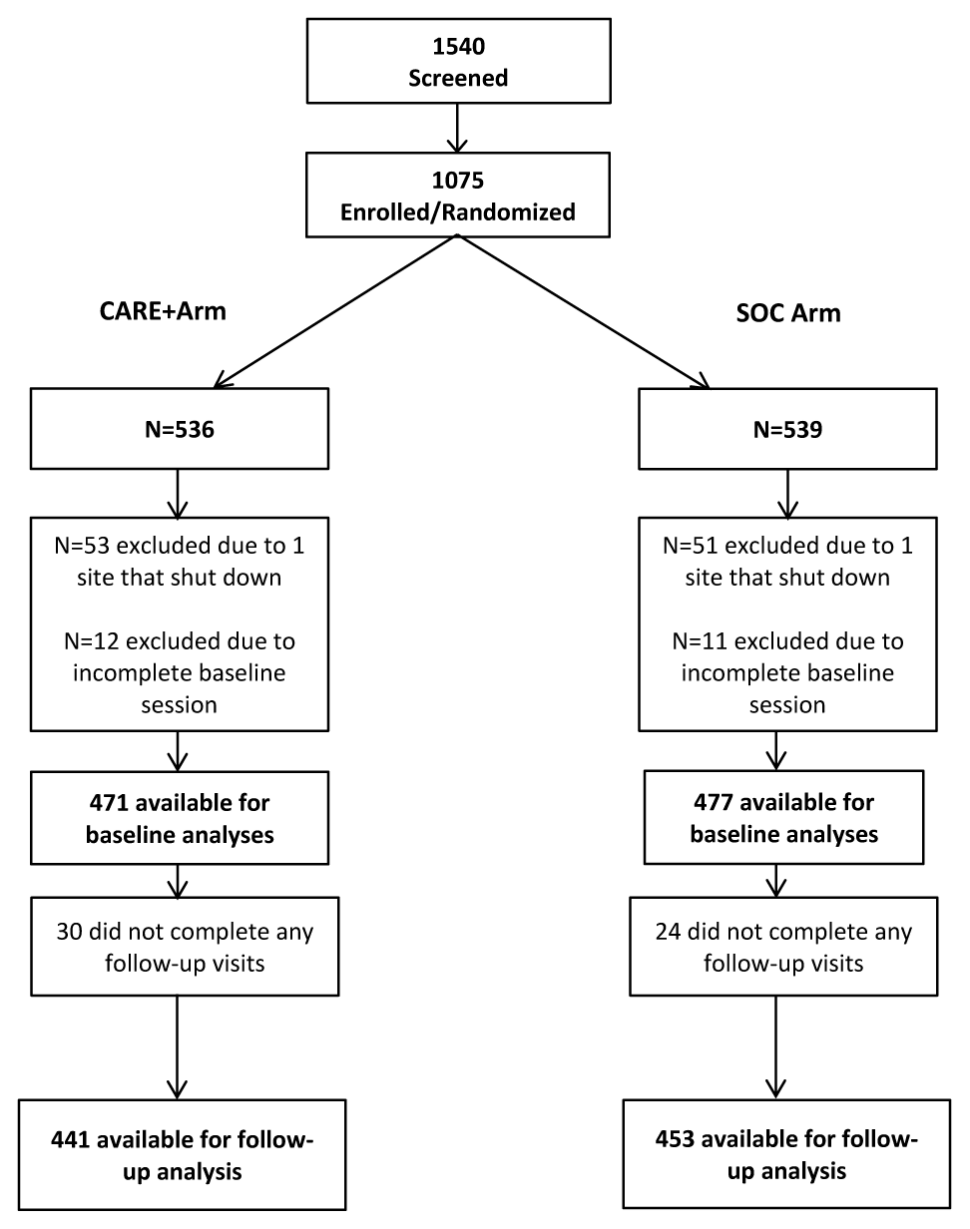
Consort diagram.

A total of 1,540 people were screened across all 11 study sites. Of those, 1,075 participants were enrolled and randomized (Figure 1). One site was terminated due to an inability to perform and 104 participants enrolled and randomized from that site were excluded from all analyses. The decision to not include these participants was made in the absence of any knowledge related to study outcomes and before many participants had completed the first follow-up visit. Additionally, 23 participants who consented but did not complete the entire baseline session and never returned to the site were considered enrolled but withdrawn from the study. Of 948 participants who were included in the baseline analyses, 54 participants did not complete any follow-up visits, resulting in a total of 894 participants included in the follow-up analysis.

Participant characteristics at baseline are presented in Table 1. A majority of participants were male (68%), identified as Black or African-American (62%) and median age was 52 (IQR 45–58). Over half of the participants (54%) identified as heterosexual while 41% identified as MSM. A large percentage of participants (41%) reported at baseline that they had not had sex in the past 3 months. Most participants (88%) were on ART at baseline. Retention at the 3 months, 6 months, 9 monthd and 12 months visits was 79%, 77%, 76%, 81%, respectively and did not significantly differ between the CARE+ and SOC arms. There were no significant demographic differences between those who were retained in the study and those who were lost to follow-up (data not shown).

**Figure 2:**
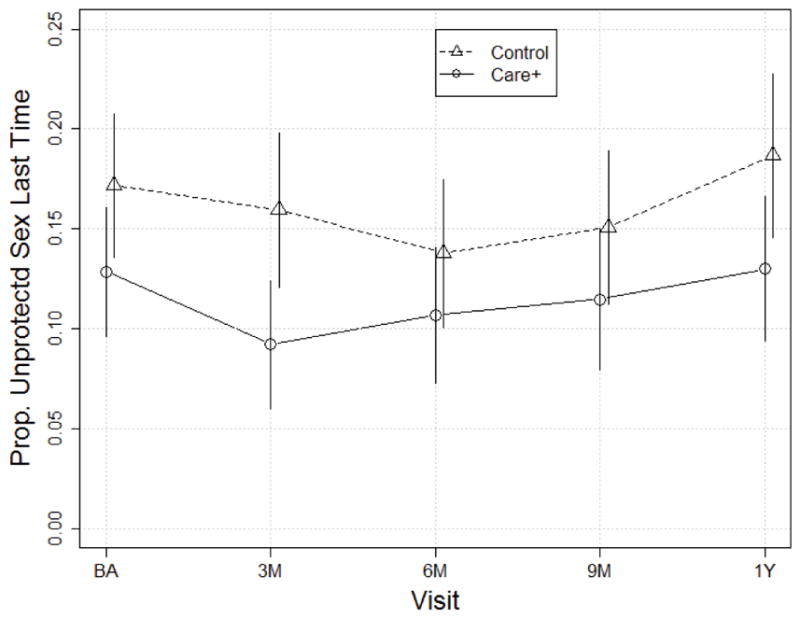
Proportion of participants reporting unprotected sex with any partner at last sex.

In the CARE+ intervention arm, the proportion of participants reporting unprotected sex with any partner was fairly low and stable over time (Figure 2), starting at 13% at baseline and finishing at 13% at month 12. The average change over time was negligible (p=0.91) (Table 2). The proportion of SOC participants reporting unprotected sex with any partner was consistently higher than in the CARE+ arm, ranging from 17% at baseline to 19% at month 12. However, the trend over time in the SOC arm was not significantly different from that among CARE+ participants (p=0.67).

**Figure 3:**
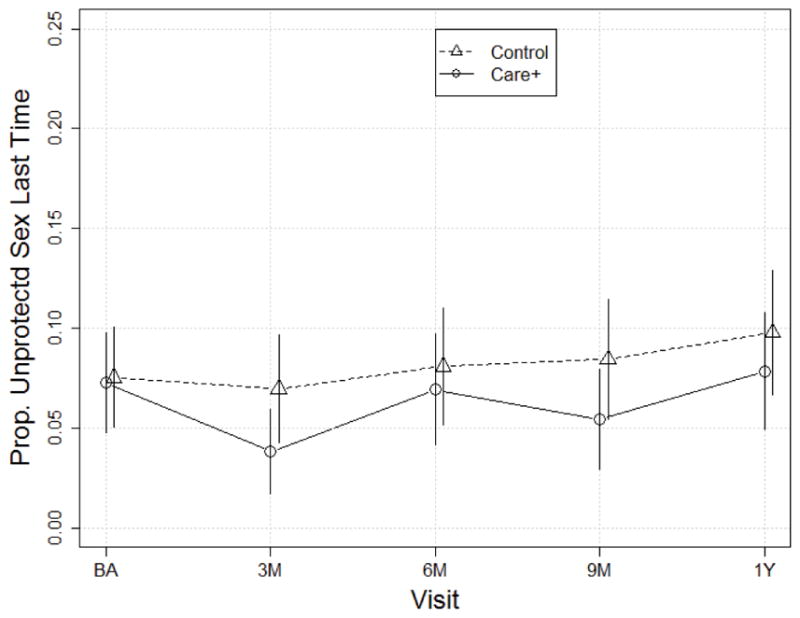
Proportion of participants reporting unprotected sex with any HIV−/unknown HIV status partner at last sex.

The findings were similar with HIV-negative or HIV-unknown-status partners for both arms: the proportion reporting unprotected sex was low and did not change significantly over time (Figure 3), ranging from 7% at baseline to 10% at Month 12, with no significant changes over time (Table 2). The proportion of SOC participants reporting unprotected sex with HIV-negative or HIV-unknown-status partners was similar to the CARE+ arm, ranging from 8% at baseline to 10% at month 12 and, again, the trend over time was not significantly different from the trend among CARE+ participants (p=0.38). Both of the above analyses were conducted separately for primary and non-primary partners, but revealed no substantive differences (data not shown).

In addition, the analysis of the composite measure produced results similar to the primary analyses results (Table 2): No significant change in self-reported risk behavior over time in the CARE+ arm (p=0.15) and no differences between the study arms (p=0.76).

The proportion of CARE+ arm participants with detectable viral load dropped slightly from 24% at baseline to 20% at month 12; however, this change over time was not statistically significant (p=0.57). The proportion of SOC arm participants with detectable viral load dropped slightly more than for the CARE+ arm, from 20% at baseline to 13% at month 12. Observed changes over time were not significantly different between the two study arms (p=0.86).

The median length of time participants in the CARE+ arm spent using the software was 50, 26, 24, 24 and 37 minutes at the baseline, 3, 6, 9 and 12 month visits, respectively. For SOC arm participants, the median length of time spent taking the risk behavior questionnaire was 41, 19, 18, 17 and 30 min at the baseline, 3, 6, 9 and 12 months visits, respectively. For participants in both arms, the longer visit times at baseline and month 12 reflect the additional survey questions asked at those time points. Overall, CARE+ arm participants watched at least one skills-building video at 61% of visits. At least one skills-building video was viewed at 67% of baseline visits and 62%, 66%, 59% and 53% at 3, 6, 9 and 12 months, respectively. Between 10 and 20 percent of participants were “flagged” for depression, intimate partner violence or suicidal ideation during the course of follow-up; however, there were no differences between study arms.

In terms of self-reported acceptability of the intervention, most participants at the month 12 Visit (85%) either strongly agreed or agreed that computer counseling on HIV prevention would be a good addition to counseling given in person by a provider. Additionally, more than half of participants (57%) at the month 12 visit either strongly agreed or agreed that a computer can be as good as a real person in providing counseling to HIV participants to prevent the spread of HIV and that it is easier to be honest when answering questions on the computer than it is when answering the same questions in person with a provider (68%).

## Discussion

In our study evaluating a tablet-based HIV prevention counseling tool for HIV prevention among HIV-positive individuals in care, we found that the CARE+ intervention was not effective at reducing self-reported HIV transmission risk behaviors compared to standard of care. The proportion of participants reporting unprotected sex did not change over time in either the SOC or CARE+ arms, regardless of the type of partner that was evaluated (any/all partners, HIV-negative or HIV-unknown-status partners).

Our results differ from those previously reported by Kurth et al. [35], which utilized the same CARE+ software but with additional ART adherence elements integrated. This may be due to differences in the study populations. Our study participants were more likely to be female (30% versus 12%) and African-American (62% vs. 28%), less likely to be MSM (41% versus 72%) and less likely to report any unprotected sex in the last 3 months (15% versus 19%) when compared to the prior study. It is possible that the CARE+ tool may be more effective with some risk populations than with others. In addition, our study population was having less unprotected sex and most were taking ART; thus, our population of participants may have perceived themselves to be at low risk for HIV transmission. Of note, a Spanish-language version of the CARE+ tool delivered to Latina women, also in HIV clinic settings, was recently published [38]. In that study, participants randomized to the Spanish CARE+ intervention had non-statistically significant lower viral loads, higher ART adherence and decreased sexual transmission risk behaviors, which may be attributed to inclusion of ART adherence elements left out of the modified CARE+ tool used in this study.

The PfP study was designed to fit as seamlessly as possible within the clinic operations. Thus, sites were encouraged to incorporate the study in a manner that would avoid disruption of services and to maximize utilization of services. However, some sites reported challenges with implementing the intervention, such as internet connectivity issues or bugs with the software that may have discouraged some participants from full engagement and, thus, limited its potential for success.

Another limitation of the study was the fact that a majority of intervention arm participants did not watch more than one skills-building video included in the CARE+ package and no qualitative data was collected to better understand why this occurred. Some literature suggests, however, that there may be differences in attention to HIV-prevention messages across racial groups, which may partially explain why participants in our study did not watch these videos as much as anticipated [39].

Additionally, retention of participants was not optimal. The study visits were scheduled for every three months; however, some participants, per standard of care practices, only needed to visit their HIV care providers every six months, and an extra trip to the clinic might be required to complete the study visit. The latter two issues may have reduced participants’ exposure in this intention to treat analysis, thus limiting the potential effectiveness of the CARE+ intervention.

While technology-based health interventions are actively being used across many health topics, one challenge that may be associated with the design and implementation of these types of interventions is the need to keep pace with technological advancements and user sophistication in the current era of social media tools. For example, the videos included in the CARE+ software were still frame shots with a narrator’s voice in the background rather than an actual video made up of moving images. While this design decision was intentional, in order for ease of use with other languages, the still frame shots were outdated. Behavioral intervention technologies, while promising, need to remain current and consistent with expectations of the users in order to maximize their potential for success [40].

Though the intervention used in this study was not effective in reducing unprotected sex, our study participants viewed computer-delivered counseling interventions favorably. Many participants felt that computer-delivered counseling on HIV prevention was a positive addition to the counseling delivered by a provider and felt that they could answer questions more honestly when using a computer than in person with a provider. Therefore, computer-delivered counseling might encourage participants to have more candid discussions about their HIV status and behaviors with their partners or providers or it may help participants improve HIV knowledge or attitudes towards condom use and other prevention methods [41].

## Conclusion

Harnessing the power of technology may be useful for HIV-positive individuals, contributing to efforts to prevent the transmission of HIV. Further efforts are needed to identify effective and feasible interventions that can build on the role of providers in HIV prevention counseling.

## Figures and Tables

**Table 1:** Demographics of study participants at baseline.

	Intervention Arm	Control Arm	Total
**Number Enrolled**	441	453	894
**Age (years)**			
Median (IQR)	51 (45, 58)	52 (44, 57)	52 (45, 58)
**Gender**			
Male	299 (68%)	308 (68%)	607 (68%)
Female	131 (30%)	136 (30%)	267 (30%)
Transgender	7 (2%)	7 (2%)	14 (2%)
Missing^1^	4 (1%)	2 (<1%)	6 (1%)
**Taking ART Medication at Baseline**	384 (87%)	403 (89%)	787 (88%)
**Race**			
American Indian or Alaska Native	4 (1%)	6 (1%)	10 (1%)
Black or African American	269 (61%)	286 (63%)	555 (62%)
White	58 (13%)	52 (11%)	110 (12%)
Other (Asian, Hawaiian, and multiracial)	90 (20%)	89 (20%)	179 (20%)
Missing^1^	20 (5%)	20 (4%)	40 (4%)
**Hispanic/Latino**	97 (22%)	84 (19%)	181 (20%)
**HIV Transmission Mode**			
MSM	173 (39%)	194 (43%)	367 (41%)
Injection drug use	6 (1%)	6 (1%)	12 (1%)
Heterosexual	249 (56%)	238 (53%)	487 (54%)
Transgender	0 (0%)	2 (<1%)	2 (<1%)
Unknown	13 (3%)	13 (3%)	26 (3%)
**Education**			
High school or less	246 (56%)	256 (57%)	502 (56%)
Associates/Bachelors Degree	139 (32%)	150 (33%)	289 (32%)
Graduate Degree	45 (10%)	40 (9%)	85 (10%)
Missing^1^	11 (2%)	7 (2%)	18 (2%)
**Household income before taxes**			
$0–$19,999	207 (47%)	216 (48%)	423 (47%)
$20,000–$49,999	101 (23%)	105 (23%)	206 (23%)
$50,000 or more	85 (19%)	92 (20%)	177 (20%)
Missing^1^	48 (11%)	40 (9%)	88 (10%)
**No sex in the past 3 months**	191 (43%)	176 (39%)	367 (41%)

1 Missing category includes “don’t know” or “refuse to answer” response categories as well as true missing data

**Table 2:** Participants HIV risk behaviors by study arm from baseline to month 12.

Contrast	Estimate Odds Ratio (95% CI)	P-Value
**No Condom Last Sex – Any Partner**		
Time trend in CARE+ Arm^1^	0.995 (0.908, 1.09)	0.909
Difference in SOC vs. CARE+ Time Trend^2^	1.03 (0.913, 1.15)	0.673
**No Condom Last Sex: HIV-Negative or Unknown HIV Status**		
Time trend in CARE+ Arm^1^	1.00 (0.876, 1.14)	0.996
Difference in SOC vs. CARE+ Time Trend^2^	1.08 (0.911, 1.28)	0.376
**Condom Problems in Last 3 Months**		
Time trend in CARE+ Arm^1^	0.917 (0.824, 1.02)	0.111
Difference in SOC vs. CARE+ Time Trend^2^	0.987 (0.851, 1.15)	0.868
**Sex Without Condom Last Time or Condom Problems in Last 3 Months (Composite Variable)**		
Time trend in CARE+ Arm^1^	0.952 (0.889, 1.02)	0.153
Difference in SOC vs. CARE+ Time Trend^2^	1.01 (0.924, 1.11)	0.758

1 Odds ratio for each 3-month time increment, in the CARE+ arm. An odds ratio significantly less than 1.0 would suggest a reduction in unprotected sex over time in the CARE+ arm

2 Ratio of SOC arm time trend to the CARE+ arm time trend. A ratio significantly larger than 1.0 would suggest that the SOC arm improved less over time than the CARE+ arm or got worse
